# Steeper medial posterior tibial slope is associated with bilateral ACL injuries: A radiographic comparison with unilateral cases

**DOI:** 10.1002/jeo2.70424

**Published:** 2025-09-04

**Authors:** Julien Druel, Paul Laidet, Apoorva Khajuria, Antoine Piercecchi, Jean‐Noël Argenson, Christophe Jacquet, Fabio Sammartino, Matthieu Ollivier

**Affiliations:** ^1^ Department of Orthopedic Surgery, Institute for Locomotion Aix‐Marseille University Marseille France; ^2^ Department of Biomechanics, APHM, CNRS, ISM, St. Marguerite Hospital, Institute for Locomotion Aix‐Marseille University Marseille France; ^3^ Department of Trauma & Orthopaedic Surgery Royal London Hospital London UK

**Keywords:** ACL reconstruction, ACL rupture, knee biomechanics, posterior tibial slope, radiographic imaging

## Abstract

**Purpose:**

The posterior tibial slope (PTS) plays a key role in knee biomechanics and may influence the risk of anterior cruciate ligament (ACL) rupture as well as the outcomes of its reconstruction. We hypothesised that a steeper medial posterior tibial slope (MPTS) would be associated with an increased risk of bilateral ACL reconstruction compared to unilateral reconstruction. This study aimed to test this hypothesis by comparing the MPTS between patients undergoing unilateral ACL reconstruction (uniACLR) and those requiring non‐simultaneous bilateral ACL reconstruction (biACLR), using radiographic imaging.

**Methods:**

This single‐centre retrospective study included 114 patients (57 uniACLR, 57 biACLR), matched by age, gender, body mass index (BMI), and presence of meniscal injury. The MPTS was measured on standardised lateral radiographs. Meniscal, cartilage, and ligamentous injuries were evaluated arthroscopically. Statistical analyses included univariate and multivariate models, with a significance threshold of *p* < 0.05.

**Results:**

Patients in the biACLR group demonstrated a significantly higher MPTS compared to the uniACLR group (mean ± SD: 12.39° ± 2.74° vs. 8.16° ± 1.67°; mean difference 4.23°, 95% confidence interval: 3.35°–5.11°; *p* < 0.0001). No significant differences were observed between groups in meniscal, cartilaginous lesions, graft selection, use of lateral extra‐articular tenodesis, or reoperation rates. Subgroup analysis indicated that higher MPTS was particularly associated with meniscal root tears.

**Conclusion:**

A steeper MPTS is significantly associated with bilateral ACL reconstruction, suggesting it may represent an anatomical risk factor for repeated ACL injuries. Systematic assessment of MPTS may help identify patients at higher risk of contralateral ACL injury, aiding in surgical planning and postoperative monitoring.

**Level of Evidence:**

Level III, retrospective comparative cohort study.

**Abbreviations**
ACL: anterior cruciate ligament
ACLR: anterior cruciate ligament reconstruction
biACLR: bilateral anterior cruciate ligament
BMI: body mass index
LET: lateral extra‐articular tenodesis
MPTS: medial posterior tibial slope
PMC: postero medial compartment
PTS: posterior tibial slope
uniACL: unilateral anterior cruciate ligament
XR‐PTS: x‐ray measurement of the posterior tibial slope

## INTRODUCTION

Anterior cruciate ligament (ACL) injuries are among the most common knee injuries, particularly in physically active individuals [12, 22]. ACL reconstruction (ACLR) remains the gold standard for restoring knee stability and function following an ACL rupture [8, 19]. However, despite advances in surgical techniques and rehabilitation, a significant proportion of patients experience graft failure or sustain a contralateral ACL injury during follow‐up [14, 21].

The posterior tibial slope (PTS) has emerged as an important anatomical factor influencing knee biomechanics [9, 17]. An increased PTS has been shown to result in greater anterior tibial translation and higher strain on the ACL, thereby predisposing individuals to ACL injury and possibly impacting outcomes of ACLR [10, 11]. Although the relationship between PTS and primary ACL rupture has been investigated, the influence of PTS on unilateral versus bilateral ACL reconstruction (uniACLR vs. biACLR) remains poorly understood [7]. Comparing patients who sustained a unilateral ACL injury requiring reconstruction with those who experienced sequential ACL injuries in both knees (necessitating bilateral ACLR) may help determine whether a steep PTS represents an underlying anatomical predisposition to repeated ligamentous injury.

This study aims to explore the potential association between PTS and the risk of bilateral ACL ruptures in patients who underwent uniACLR or non‐simultaneous biACLR. We hypothesise that a steeper PTS may be linked to an increased risk of bilateral ACL injury. To address this hypothesis, we conducted a retrospective analysis using radiographic imaging to measure the medial posterior tibial slope (MPTS) in a cohort of matched patients.

By better understanding the role of PTS in ACL rupture risk, this study aims to provide evidence for the potential importance of systematic radiographic assessment of PTS in preoperative planning and risk stratification.

## METHODS

This retrospective, single‐centre observational study was conducted using data from a prospectively maintained institutional database. The study protocol was approved by the institutional review board (IRB approval number: PADS22‐182), and all procedures were performed in accordance with the Declaration of Helsinki.

We identified all patients who underwent primary isolated ACL reconstruction for a complete ACL rupture at our institution between February 2016 and April 2024. Inclusion criteria were: (1) patients aged 15–80 years; (2) undergoing unilateral or non‐simultaneous bilateral ACL reconstruction (biACLR); and (3) standardised lateral knee radiographs performed within three months of injury. Exclusion criteria were: (1) multiligamentous injuries requiring surgery; (2) associated fractures; (3) inadequate radiographs (e.g., insufficient condylar overlap); (4) prior ACL reconstruction, graft failure, or other previous knee surgery; and (5) major prior knee trauma.

From 750 eligible patients, 533 met inclusion criteria. Among these, all patients with biACLR and adequate imaging (*n* = 57) were identified. For comparison, 57 uniACLR patients were selected from the remaining cohort using 1:1 matching based on age, sex, body mass index (BMI), and presence of meniscal injury. The matching process only assessed whether a meniscal injury was present or not, further subtyping of the meniscal injury was not carried out at this stage. The matching process was performed manually, without propensity scores, to ensure balanced groups with similar baseline characteristics.

**Figure 1:**
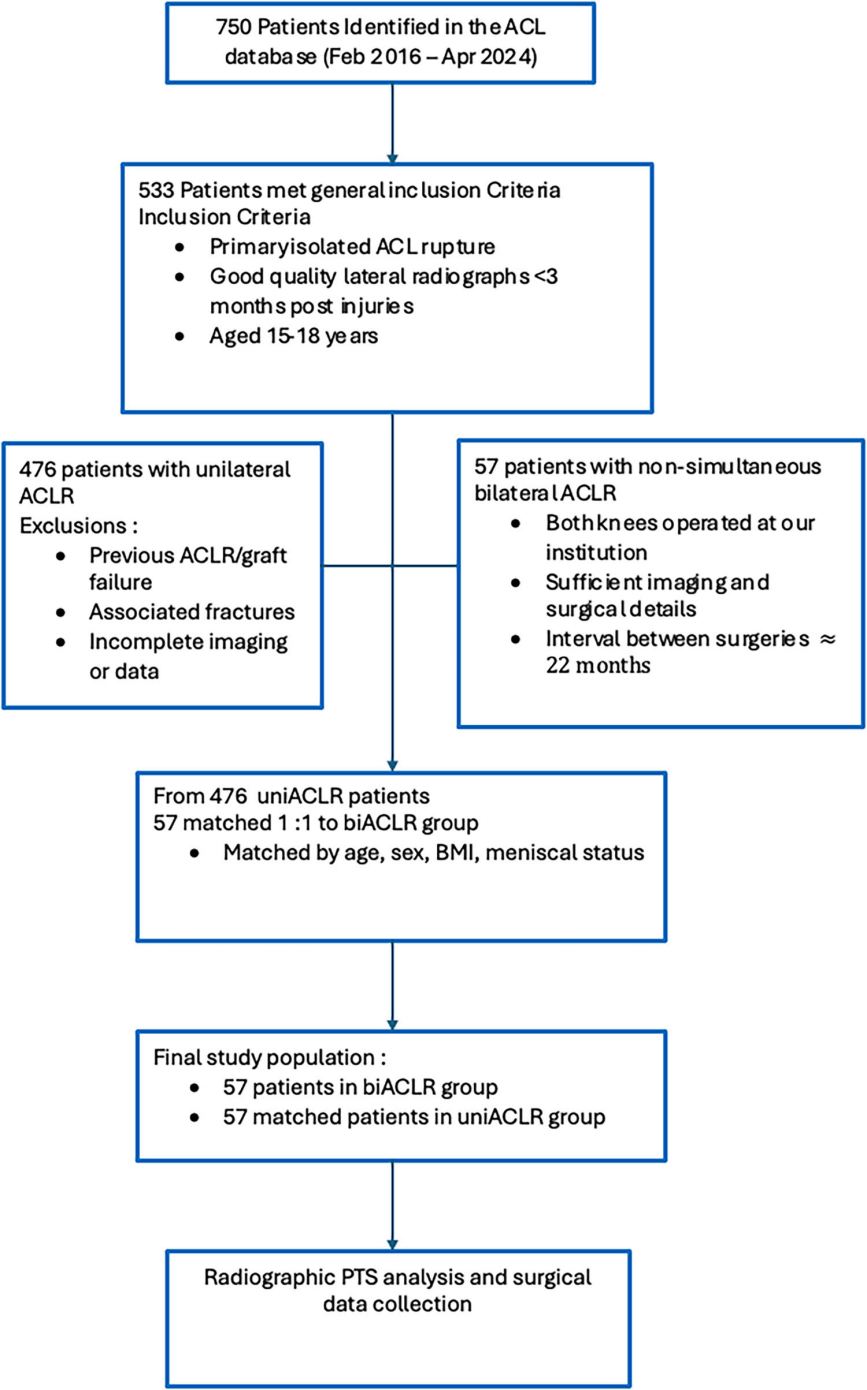
A flow diagram illustrating patient's enrolment. ACL, anterior cruciate ligament; ACLR, anterior cruciate ligament reconstruction; biACLR, bilateral anterior cruciate ligament; PTS, posterior tibial slope.

All biACLR patients had undergone two distinct ACL reconstructions, one on each knee, both performed at our institution and within the defined study period. The average time interval between the two surgeries in the biACLR group was approximately 12–24 months, ensuring exposure to consistent surgical protocols. The final sample included 114 patients divided equally between the two groups (Figure 1).

To further explore the relationship between PTS and intra‐articular pathology, a subgroup analysis was conducted according to the type of meniscal injury. Medial and lateral meniscal injuries were classified arthroscopically into five categories: no lesion, radial, vertical, ramp (for Medial meniscus only), and root tears. For each lesion type, the average preoperative MPTS was calculated using radiographic measurements as described in the next paragraph. This allowed for the assessment of potential associations between specific type of meniscal injuries and tibial slope morphology (Table 1).

**Table 1:** Patient characteristics by group (uniACLR vs. biACLR).

Variable	uniACLR (*n* = 57)	biACLR (*n* = 57)
Age (mean ± SD)	27.3 ± 7.8	27.3 ± 7.7
Sex (male)	24.6%	24.6%
Posterior tibial slope (preop)	8.16° ± 1.67	12.39° ± 2.74
Posterior tibial slope (postop)	8.14° ± 1.70	12.39° ± 2.74
Meniscal lesion (any)	~75% (43)	~81% (46)
• Medial meniscus lesion	42.6% (23)	52.6% (31)
• Lateral meniscus lesion	45.6% (26)	45.6% (26)
• Ramp lesion	7	9
• Root lesion	9	14
Cartilage lesion (any)	~10% (6)	~10% (6)
LET performed	22.8% (13)	36.8% (21)
Reoperation	5.3% (3)	5.3% (3)
Revision (meniscus or ACL)	0%	5.3% (3)

Abbreviations: biACLR, bilateral anterior cruciate ligament reconstruction; LET, lateral extra‐articular tenodesis; SD, standard deviation; uniACLR, unilateral anterior cruciate ligament reconstruction.

### Clinical and arthroscopic analysis

All patients underwent knee arthroscopy independently by three senior orthopaedic surgeons. An assessment of meniscal, cartilage, and ligament injuries was performed. Meniscal tears were classified as radial tear, vertical tear, ramp lesion, and root tear. The posteromedial compartment (PMC) was systematically examined using transnotch arthroscopy to identify posteromedial meniscal injuries. A posteromedial needle test was conducted during each procedure to detect the presence of ramp lesions. Meniscal repair was performed when feasible using sutures or root reinsertion. ACL reconstruction was carried out using various grafts, including hamstring tendon, quadriceps tendon, and allograft. A lateral extra‐articular tenodesis (LET) procedure was performed to repair the anterolateral ligament using the Lemaire technique or with a Gracilis autograft.

### Radiological analysis

Accurate differentiation of the lateral plateau is often challenging due to projection limitations on plain radiographs, therefore, the MPTS was measured independently. All images were accessed through a picture archiving and communication system (PACS), and radiographic measurements were performed using the Centricity PACS software.

All measurements were performed by a single experienced orthopedic surgeon blinded to group allocation. Intra‐rater reliability was assessed by repeating measurements on 10% of randomly selected images after two weeks, yielding an intraclass correlation coefficient (ICC) of 0.91. Inter‐rater reliability was assessed postoperatively on a separate random sample, also yielding an ICC >  0.90. Intra‐rater reliability yielded an ICC of 0.91 with a standard error of measurement (SEM) of 0.32°. Inter‐rater reliability showed an ICC of 0.90 with a SEM of 0.35.

**Figure 2:**
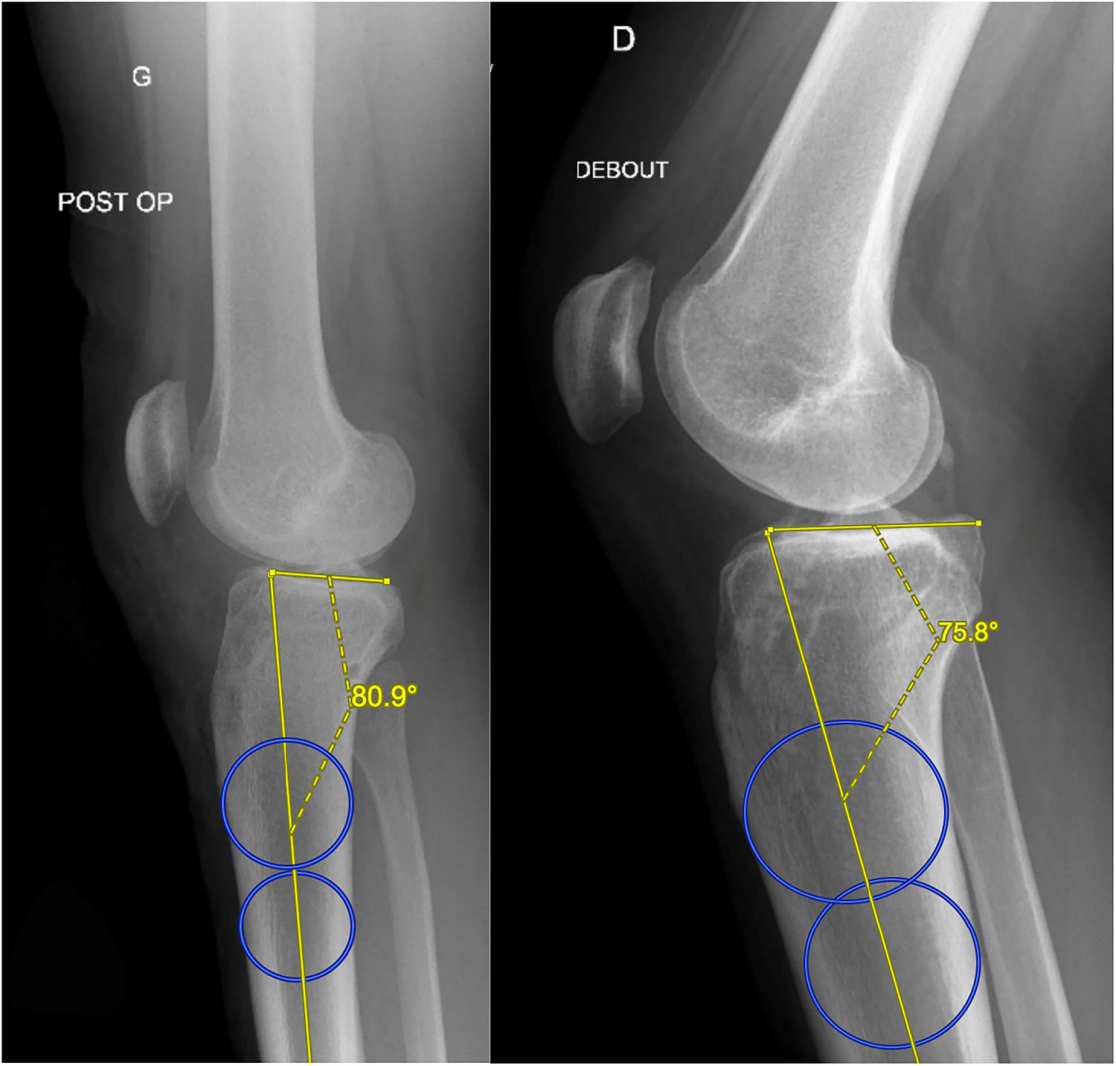
Lateral Knee Radiograph demonstrating the two‐circle method in measuring the medial posterior tibial slope (MPTS) in the uniACLR group (on the left) and biACLR group (on the right). biACLR, bilateral anterior cruciate ligament reconstruction; uniACLR, unilateral anterior cruciate ligament reconstruction.

Several techniques exist for radiographic or X‐ray measurement of the posterior tibial slope (XR‐PTS), most of which position the proximal reference point just below the tibial tubercle to reduce the effect of metaphyseal flare on axis alignment. [13] The main variability among methods lies in the placement of the distal reference point. In this study, the distal reference point was set as far distal as possible, maintaining a minimum 5 cm distance between the proximal and distal points. The proximal tibial axis was identified using a circle‐fitting method, with the circle′s centre serving as the reference. XR‐PTS was determined by subtracting the angle between this axis and a line connecting the anterior and posterior points of the medial tibial plateau from 90°, a proven and reliable approach [3] (Figure 2).

Radiograph quality was evaluated based on three criteria: posterior condyle overlap (< 5 mm), distal condyle overlap (< 5 mm), and a minimum 5 cm distance between proximal and distal reference points.

### Statistical analysis

Data were analysed using SPSS Statistics version 22.0 (IBM Corp., Armonk, NY, USA). Sample size was calculated to detect a mean difference in MPTS of at least 3°, which exceeds the standard deviation (SD) observed in previous studies (approximately 2.0°). Assuming a standard deviation of 3.0°, a two‐sided alpha of 0.05, and a power of 80%, at least 53 patients per group were required [18].

Descriptive statistics were used to summarise the characteristics of the study population, including means, SD, and confidence intervals (CI) for continuous variables, and frequencies and percentages for categorical variables.

Normality of continuous data distributions was assessed using the Shapiro–Wilk test. Given that the primary variable of interest (MPTS) followed a normal distribution, group comparisons between the bilateral ACL reconstruction (biACLR) and unilateral ACL reconstruction (uniACLR) groups were performed using independent‐samples Student′s *t*‐tests. When data were not normally distributed, non‐parametric alternatives such as the Mann–Whitney *U* test were applied. For categorical variables (e.g., sex, presence of meniscal tear, cartilage damage, graft type, and LET), chi‐square tests or Fisher′s exact tests were used depending on cell counts.

To evaluate the association between MPTS and group allocation (biACLR vs. uniACLR), a one‐way analysis of variance (ANOVA) was initially performed, followed by multivariate linear regression to adjust for potential confounding factors (age, sex, BMI and presence of meniscal lesions). The regression model reported unstandardised coefficients (β), 95% CI, and *p*‐values.

Subgroup analyses were conducted to assess the relationship between MPTS and specific meniscal tear patterns (radial, vertical, ramp, root or none). Comparisons of slope values across meniscal injury subtypes were analysed using one‐way ANOVA or Kruskal–Wallis tests, as appropriate.

All tests were two‐tailed, and a *p*‐value < 0.05 was considered statistically significant. No imputation was performed for missing data.

This study adheres to the STROBE (Strengthening the Reporting of Observational Studies in Epidemiology) guidelines; the checklist can be provided as a supplementary file upon request.

## RESULTS

A total of 114 patients were included in the analysis, with 57 having undergone unilateral ACL reconstruction (uniACLR) and 57 bilateral ACL reconstruction (biACLR). The two groups were matched for age, sex, BMI and presence of meniscal injury. A summary of patient demographics is provided in Table 1. No statistically significant differences were identified between the groups in terms of age distribution, sex, or BMI. Similarly, graft selection, the addition of a LET procedure and rates of reoperation were comparable between the two groups.

The baseline characteristics of the two groups (uniACLR and biACLR) are presented in Table 2.

**Table 2:** Relationship between meniscal lesion types and average preoperative posterior tibial slope measured on standardised radiographs.

Lateral meniscus injuries	The average of the preoperative slope (°)
No lesion	9.0
Radial lesion	9.1
Vertical lesion	8.6
Root lesion	11.2

MediaI meniscus injuries	The average of the preoperative slope (°)
No lesion	8.7
Radial lesion	8.7
Vertical lesion	9.1
Ramp lesion	9.1
Root lesion	10.0

**Figure 3:**
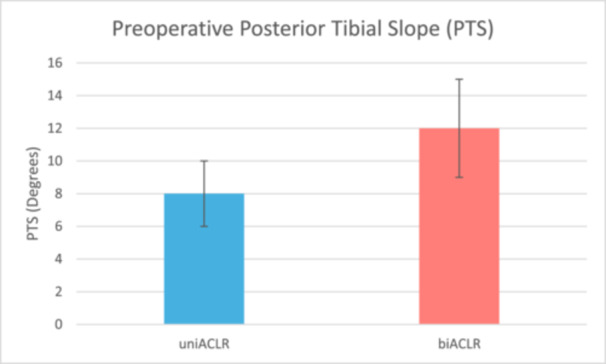
Comparison of preoperative posterior tibial slope (PTS) between uniACLR and biACLR Groups. biACLR, bilateral anterior cruciate ligament reconstruction; uniACLR, unilateral anterior cruciate ligament reconstruction.

Analysis of preoperative MPTS revealed significantly higher values in the biACLR group (mean ± SD: 12.39° ± 2.74°) compared to the uniACLR group (8.16° ± 1.67°), with a mean difference of 4.23° (95% CI: 3.35°–5.11°; *p *< 0.0001). These findings were consistent in postoperative measurements as well (Figure 3). The difference remained significant in a multivariate linear regression model adjusted for age, sex, BMI and meniscal injury (*β* = 3.98°, 95% CI: 2.91°–5.05°; *p* <  0.0001).

**Figure 4:**
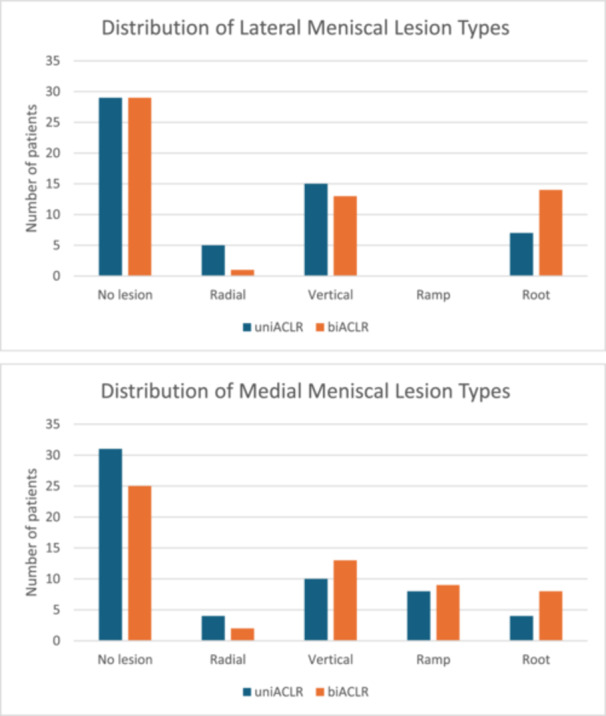
Distribution of medial and lateral meniscal lesion types in uniACLR versus biACLR patients. biACLR, bilateral anterior cruciate ligament reconstruction; uniACLR, unilateral anterior cruciate ligament reconstruction.

The distribution and frequency of medial and lateral meniscal injuries, including ramp and root lesions, did not differ significantly between the groups. No significant differences were found in cartilage lesion rates across compartments (Figure 4).

Subgroup analyses of meniscal injuries showed that patients with meniscal root tears had the highest average preoperative MPTS (lateral root tear: 11.2° ± 1.9°; medial root tear: 10.0° ± 1.7°), compared to those with radial, vertical, or ramp tears (all below 9.2°). The association between root tears and higher MPTS was statistically significant (*p* = 0.008, ANOVA) (Table 1).

Out of all participants, six patients (5.3%) required a subsequent surgical intervention. Three of these were due to failed meniscal repairs, while the other three involved ACL graft failure and required revision ACL reconstruction. These events occurred equally between the groups and were descriptively reported without significant statistical difference.

In summary, our findings demonstrate a significant association between steeper MPTS and bilateral ACL reconstruction, independent of age, sex, BMI and meniscal injury, while other intra‐articular injuries and surgical variables were similar between groups.

## DISCUSSION

The main finding of this study was that patients undergoing biACLR exhibit a significantly higher MPTS compared to those who required only unilateral reconstruction. These results support our initial hypothesis that a steeper MPTS is associated with an increased risk of bilateral ACL injury.

This finding is consistent with previous biomechanical and clinical studies demonstrating that increased tibial slope is associated with greater anterior tibial translation and higher strain on the ACL, thereby predisposing individuals to injury and graft failure. Several studies have highlighted that patients undergoing ACL revision or re‐rupture often present with a steeper PTS compared to those undergoing primary ACL reconstruction [1, 4].

Dean et al. demonstrated in a systematic review and meta‐analysis that an increased PTS is significantly more frequent in patients with failed ACL reconstructions compared to primary tears or intact ACLs [4]. Similarly, Beel et al. emphasised the high prevalence of increased PTS in ACL revision patients, recommending a patient‐specific surgical approach to address this anatomical risk factor [1]. In particular, our results align with the work of Garra et al., who also reported an association between steeper posterior tibial slope and bilateral ACL injuries [7]. However, our study adds to the literature by relying on radiographic measurements alone a widely accessible modality and by providing a detailed analysis of meniscal injury subtypes, revealing a specific association between high MPTS and meniscal root tears.

In our study root tears were associated with the highest preoperative MPTS, particularly on the lateral side (11.2°). This is consistent with biomechanical data showing that root tears disrupt the meniscal hoop mechanism, increasing tibiofemoral contact forces. When combined with a steep tibial slope, this creates a cumulative mechanical overload that may predispose to both ACL rupture and severe meniscal lesions [6, 11, 16]. These findings suggest that elevated MPTS is not only a predictor of ligamentous injury, but may also be associated with higher‐grade, intra‐articular damage, such as root lesions. This interplay reinforces the importance of systematic radiographic slope assessment, especially in young, active patients or those with a history of contralateral injury. It may also justify a more comprehensive surgical approach, including root repair and, in select cases, slope‐correcting osteotomy.

Although the groups in our study were well matched for age, BMI, meniscal injury, and gender, PTS emerged as a statistically significant differentiating factor. This reinforces the hypothesis that anatomical features may act independently of other intra‐articular variables in influencing ACL injury patterns and reconstruction outcomes.

Contrary to some earlier studies, our data did not show differences in graft selection, use of LET procedure, or reoperation rates between the uniACLR and biACLR groups. However, slope‐correcting osteotomies may play a preventive role in high‐risk patients, particularly in revision settings. Rozinthe et al. demonstrated that such interventions can result in favourable long‐term outcomes and reduced risk of re‐rupture [20].

Finally, Dejour et al. have shown that PTS is associated with increased preoperative laxity in ACL‐deficient knees, further highlighting its relevance in pre‐surgical evaluation and planning [5].

Our findings underscore the potential clinical relevance of assessing MPTS as part of preoperative evaluation in patients undergoing ACL reconstruction. While MPTS is a non‐modifiable anatomical factor, its identification may help stratify the risk of contralateral ACL injury [14, 21]. Systematic radiographic assessment of PTS after a first ACL injury could help identify patients with a structural predisposition and prompt individualised risk reduction protocols. Such strategies might include tailored rehabilitation with neuromuscular training, delayed return‐to‐sport, closer clinical monitoring, or even preemptive consideration of slope‐reducing osteotomy in exceptional cases, particularly in young athletes with additional risk factors [2, 15, 20]. Although speculative at this stage, these considerations may serve as a basis for future prospective studies aiming to evaluate whether preventive strategies tailored to anatomical risk factors can reduce the incidence of secondary ACL injuries.

Our study has several limitations. First, the retrospective, single‐centre design may limit the generalisability of our findings. Second, although groups were matched for key variables (age, sex, BMI and meniscal injury), unmeasured confounding factors such as injury mechanism, rehabilitation adherence, or neuromuscular control could still influence outcomes. Third, only the medial tibial slope was measured, as reliable measurement of the lateral slope on radiographs is technically challenging; yet, the lateral slope may also contribute to ACL injury risk [9, 10]. Fourth, we did not assess functional outcomes, return‐to‐sport rates, or long‐term graft survival. Finally, this study demonstrates an association between MPTS and bilateral ACL reconstruction but cannot establish causation.

Despite these limitations, our findings underscore the importance of recognising steep MPTS as a potential anatomical risk factor for bilateral ACL injuries. Systematic assessment of MPTS may help clinicians identify patients at higher risk of contralateral ACL injury and may inform surgical planning and patient counselling. Further prospective studies are needed to confirm these findings and to determine whether addressing tibial slope in high‐risk patients improves clinical outcomes.

## CONCLUSION

A steeper MPTS was significantly associated with bilateral ACLR compared to unilateral reconstruction in our matched cohort. These findings suggest that increased MPTS may represent an anatomical risk factor for repeated ACL injuries. Systematic assessment of MPTS during preoperative evaluation may help identify patients at higher risk of contralateral ACL injury and assist in surgical planning and patient counselling. Further prospective studies are needed to confirm these findings and to determine whether addressing MPTS can improve clinical outcomes.

## AUTHOR CONTRIBUTIONS

Julien Druel, Paul Laidet, Fabio Sammartino, Apoorva Khajuria and Matthieu Ollivier contributed to study conception and design. Julien Druel, Paul Laidet, Fabio Sammartino and Antoine Piercecchi collected and analysed the data. Julien Druel and Christophe Jacquet performed the radiographic measurements. Jean‐Noël Argenson and Matthieu Ollivier supervised the study. Julien Druel drafted the manuscript. All authors revised the manuscript critically for important intellectual content and approved the final version.

## CONFLICT OF INTEREST STATEMENT

The authors declare no conflicts of interest.

## ETHICS STATEMENT

Please include the name of the institutional review board (IRB) and the approval number. If not applicable, please state so.: This study was approved by the local Institutional Review Board (Comité d′éthique de l′Institut du Mouvement et de l′Appareil Locomoteur, Aix‐Marseille Université) under approval number IRB‐2023‐ACL‐07.

## Data Availability

The data that support the findings of this study are available from the corresponding author upon reasonable request. Due to institutional and ethical restrictions, the data are not publicly available.

## References

[1] Beel W , Schuster P , Michalski S , Mayer P , Schlumberger M , Hielscher L , et al. High prevalence of increased posterior tibial slope in ACL revision surgery demands a patient‐specific approach. Knee Surg Sports Traumatol Arthrosc. 2023;31(7):2974–2982. 36622421 10.1007/s00167-023-07313-2

[2] Buckthorpe M . Optimising the late‐stage rehabilitation and return‐to‐sport training and testing process after ACL reconstruction. Sports Med. 2019;49(7):1043–1058. 31004279 10.1007/s40279-019-01102-z

[3] Chen Y , Ding J , Dai S , Yang J , Wang M , Tian T , et al. Radiographic measurement of the posterior tibial slope in normal Chinese adults: a retrospective cohort study. BMC Musculoskelet Disord. 2022;23(1):386. 35473639 10.1186/s12891-022-05319-4 PMC9040249

[4] Dean RS , DePhillipo NN , LaPrade RF . Posterior tibial slope in patients with torn acl reconstruction grafts compared with primary tear or native ACL: a systematic review and meta‐analysis. Orthop J Sports Med. 2022;10(4):23259671221079380. 35425846 10.1177/23259671221079380 PMC9003651

[5] Dejour D , Pungitore M , Valluy J , Nover L , Saffarini M , Demey G . Preoperative laxity in ACL‐deficient knees increases with posterior tibial slope and medial meniscal tears. Knee Surg Sports Traumatol Arthrosc. 2019;27(2):564–572. 30269166 10.1007/s00167-018-5180-3

[6] Dzidzishvili L , Allende F , Allahabadi S , Mowers CC , Cotter EJ , Chahla J . Increased posterior tibial slope is associated with increased risk of meniscal root tears: a systematic review. Am J Sports Med. 2024;52(13):3427–3435. 38362610 10.1177/03635465231225981

[7] Garra S , Li ZI , Triana J , Rao N , Alaia MJ , Strauss EJ , et al. Posterior tibial slope in patients undergoing bilateral versus unilateral ACL reconstruction: MRI and radiographic analyses. Am J Sports Med. 2023;51(9):2275–2284. 38073181 10.1177/03635465231177086

[8] van der Graaff SJA , Meuffels DE , Bierma‐Zeinstra SMA , van Es EM , Verhaar JAN , Eggerding V , et al. Why, when, and in which patients nonoperative treatment of anterior cruciate ligament injury fails: an exploratory analysis of the COMPARE trial. Am J Sports Med. 2022;50(3):645–651. 35048733 10.1177/03635465211068532

[9] Hohmann E , Tetsworth K , Glatt V , Ngcelwane M , Keough N . Medial and lateral posterior tibial slope are independent risk factors for noncontact ACL injury in both men and women. Orthop J Sports Med. 2021;9(8):23259671211015940. 34409110 10.1177/23259671211015940 PMC8366133

[10] Kolbe R , Schmidt‐Hebbel A , Forkel P , Pogorzelski J , Imhoff AB , Feucht MJ . Steep lateral tibial slope and lateral‐to‐medial slope asymmetry are risk factors for concomitant posterolateral meniscus root tears in anterior cruciate ligament injuries. Knee Surg Sports Traumatol Arthrosc. 2019;27(8):2585–2591. 30390134 10.1007/s00167-018-5279-6

[11] Liu Z , Jiang J , Yi Q , Teng Y , Liu X , He J , et al. An increased posterior tibial slope is associated with a higher risk of graft failure following ACL reconstruction: a systematic review. Knee Surg Sports Traumatol Arthrosc. 2022;30(7):2377–2387. 35124715 10.1007/s00167-022-06888-6

[12] Lyman S , Koulouvaris P , Sherman S , Do H , Mandl LA , Marx RG . Epidemiology of anterior cruciate ligament reconstruction: trends, readmissions, and subsequent knee surgery. J Bone Jt Surg‐Am. 2009;91(10):2321–2328. 10.2106/JBJS.H.00539 19797565

[13] Mabrouk A , An J‐S , Fernandes LR , Kley K , Jacquet C , Ollivier M . Maintaining posterior tibial slope and patellar height during medial opening wedge high tibial osteotomy. Orthop J Sports Med. 2023;11(12):23259671231213595. 38090657 10.1177/23259671231213595 PMC10714891

[14] Magnussen RA , Meschbach NT , Kaeding CC , Wright RW , Spindler KP . ACL graft and contralateral ACL tear risk within ten years following reconstruction: a systematic review. JBJS Reviews. 2015;3(1):e3. 10.2106/JBJS.RVW.N.00052 27501023

[15] van Melick N , van Cingel REH , Brooijmans F , Neeter C , van Tienen T , Hullegie W , et al. Evidence‐based clinical practice update: practice guidelines for anterior cruciate ligament rehabilitation based on a systematic review and multidisciplinary consensus. Br J Sports Med. 2016;50(24):1506–1515. 27539507 10.1136/bjsports-2015-095898

[16] Melugin HP , Brown JR , Hollenbeck JFM , Fossum BW , Whalen RJ , Ganokroj P , et al. Increased posterior tibial slope increases force on the posterior medial meniscus root. Am J Sports Med. 2023;51(12):3197–3203. 37715505 10.1177/03635465231195841

[17] Micicoi G , Jacquet C , Khakha R , LiArno S , Faizan A , Seil R , et al. Femoral and tibial bony risk factors for anterior cruciate ligament injuries are present in more than 50% of healthy individuals. Am J Sports Med. 2021;49(14):3816–3824. 34710345 10.1177/03635465211050421

[18] Pangaud C , Laumonerie P , Dagneaux L , LiArno S , Wellings P , Faizan A , et al. Measurement of the posterior tibial slope depends on ethnicity, sex, and lower limb alignment: a computed tomography analysis of 378 healthy participants. Orthop J Sports Med. 2020;8(1):2325967119895258. 32047827 10.1177/2325967119895258 PMC6984458

[19] Papaleontiou A , Poupard AM , Mahajan UD , Tsantanis P . Conservative vs surgical treatment of anterior cruciate ligament rupture: a systematic review. Cureus. 2024;16(3):e56532. 38646275 10.7759/cureus.56532 PMC11027445

[20] Rozinthe A , van Rooij F , Demey G , Saffarini M , Dejour D . Tibial slope correction combined with second revision ACLR grants good clinical outcomes and prevents graft rupture at 7‐15‐year follow‐up. Knee Surg Sports Traumatol Arthrosc. 2022;30(7):2336–2341. 34842944 10.1007/s00167-021-06750-1

[21] Sanders TL , Pareek A , Hewett TE , Levy BA , Dahm DL , Stuart MJ , et al. Long‐term rate of graft failure after ACL reconstruction: a geographic population cohort analysis. Knee Surg Sports Traumatol Arthrosc. 2017;25(1):222–228. 27522592 10.1007/s00167-016-4275-y

[22] Shea KG , Grimm NL , Ewing CK , Aoki SK . Youth sports anterior cruciate ligament and knee injury epidemiology: who is getting injured? In what sports? When? Clin Sports Med. 2011;30(4):691–706. 22018311 10.1016/j.csm.2011.07.004

